# En bloc resection of bladder tumour: the rebirth of past through reminiscence

**DOI:** 10.1007/s00345-023-04547-0

**Published:** 2023-08-16

**Authors:** Jeremy Yuen-Chun Teoh, David D’Andrea, Andrea Gallioli, Takafumi Yanagisawa, Steven MacLennan, Rossella Nicoletti, Ng Chi Fai, Davide Maffei, Rodolfo Hurle, Lukas Lusuardi, Bernard Malavaud, Jun Miki, Mario Kramer, Hugh Mostafid, Dmitry Enikeev, Marek Babjuk, Alberto Breda, Shahrokh Shariat, Paolo Gontero, Thomas Herrmann

**Affiliations:** 1 Department of Surgery, S.H. Ho Urology Centre, The Chinese University of Hong Kong, Hong Kong SAR, China; 2 Urothelial Cancer Working Group, European Association of Urology-Young Academic Urologists (EAU-YAU, Amsterdam, Netherlands; 3 Department of Urology, Comprehensive Cancer Center, Medical University of Vienna, Vienna, Austria; 4 Department of Urology, Fundació Puigvert, Autonoma University of Barcelona, Barcelona, Spain; 5 Department of Urology, The Jikei University School of Medicine, Tokyo, Japan; 6 Academic Urology Unit, University of Aberdeen, Aberdeen, UK; 7 Unit of Urological Robotic Surgery and Renal Transplantation, Careggi Hospital, University of Florence, Florence, Italy; 8 Department of Urology, University College London Hospital NHS Foundation Trust, London, UK; 9 Department of Biomedical Sciences, Humanitas University, Milan, Italy; 10 Department of Urology, IRCCS Humanitas Research Hospital, Rozzano, Italy; 11 Department of Urology and Andrology, Salzburg University Hospital, Paracelsus Medical University, 5020 Salzburg, Austria; 12 Institut Universitaire du Cancer Toulouse-Oncopôle, Toulouse, France; 13 Department of Urology, University Hospital Schleswig-Holstein, Lübeck, Germany; 14 Department of Urology, The Stokes Centre for Urology, Royal Surrey Hospital, Guildford, UK; 15 Institute for Urology and Reproductive Health, Sechenov University, Moscow, Russia; 16 Department of Urology, 2nd Faculty of Medicine, Hospital Motol, Charles University, Prague, Czech Republic; 17 Department of Urology, Weill Cornell Medical College, New York, NY USA; 18 Department of Urology, University of Texas Southwestern, Dallas, TX USA; 19 Hourani Center for Applied Scientific Research, Al-Ahliyya Amman University, Amman, Jordan; 20 Department of Urology, Città Della Salute e Della Scienza, University of Torino School of Medicine, Turin, Italy; 21 Department of Urology, Kantonsspital Frauenfeld, Frauenfeld, Switzerland

**Keywords:** Bladder cancer, En bloc resection, ERBT, Transurethral resection, TURBT, NMIBC

## Abstract

**Purpose:**

To learn about the history and development of en bloc resection of bladder tumour (ERBT), and to discuss its future directions in managing bladder cancer.

**Methods:**

In this narrative review, we summarised the history and early development of ERBT, previous attempts in overcoming the tumour size limitation, consolidative effort in standardising the ERBT procedure, emerging evidence in ERBT, evolving concepts in treating large bladder tumours, and the future directions of ERBT.

**Results:**

Since the first report on ERBT in 1980, there has been tremendous advancement in terms of its technique, energy modalities and tumour retrieval methods. In 2020, the international consensus statement on ERBT has been developed and it serves as a standard reference for urologists to practise ERBT. Recently, high-quality evidence on ERBT has been emerging. Of note, the EB-StaR study showed that ERBT led to a reduction in 1-year recurrence rate from 38.1 to 28.5%. An individual patient data meta-analysis is currently underway, and it will be instrumental in defining the true value of ERBT in treating non-muscle-invasive bladder cancer. For large bladder tumours, modified approaches of ERBT should be accepted, as the quality of resection is more important than a mere removal of tumour in one piece. The global ERBT registry has been launched to study the value of ERBT in a real-world setting.

**Conclusion:**

ERBT is a promising surgical technique in treating bladder cancer and it has gained increasing interest globally. It is about time for us to embrace this technique in our clinical practice.

## Introduction

The development of transurethral resection of bladder tumour (TURBT) dates back to 1806, when Bozzini invented the Lichtleiter, a speculum with a candle and a mirror, which allowed visualisation of internal body cavities [1]. In 1877, the very first direct-vision cystoscope was developed and introduced by Nitze [2]. Apart from having a clear endoscopic vision, a reliable energy source is needed for fulguration and resection of bladder tumours. In 1908, Wappler developed a resonator which could generate monopolar current, but it was in 1910 when Beer reported its use for electrocoagulation of bladder tumours [3]. In 1926, Stern introduced the first resectoscope [4], consisting of a sheath and working parts including a direct-vision telescope, a light carrier, a water conduit and an active electrode. In 1931, McCarthy further improved the resectoscope by incorporating separate currents for coagulation and cutting, and introducing an active working element which allows tumour resection from far end towards the endoscope [4]. The Stern-McCarthy resectoscope becomes the foundation of TURBT, which has remained as the cornerstone treatment of bladder cancer until now [5].

TURBT is no doubt a revolutionary invention in treating bladder cancer. However, there are two main limitations with the procedure. First, bladder tumour is actively fragmented during TURBT. It results in floating tumour cells which may re-implant to the bladder wall and lead to early disease recurrence [6, 7]. Second, whether a complete resection has been achieved is totally dependent on the surgeon’s experience and judgement. Unfortunately, this is prone to error and residual disease can occur despite a ‘complete’ TURBT [6, 7]. There is a constant search of a procedure that can uphold basic oncological principles, and this sets the scene for the birth of en bloc resection of bladder tumour (ERBT).

### The birth and early development of en bloc resection

**Fig. 1:**
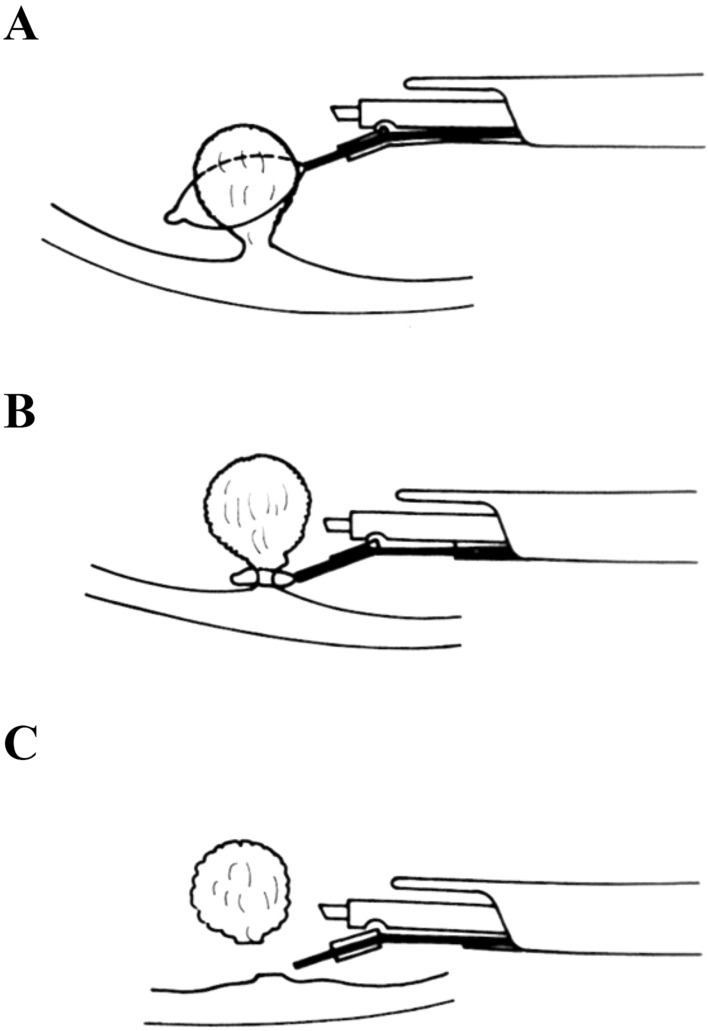
**A** Snare is opened. **B** Opened snare at basement of tumour as close as possible. **C** Tumour resected with closed snare. Adapted from Kitamura et al. J Urol 124 (6):808–809, with permission from the Journal of Urology

**Fig. 2:**
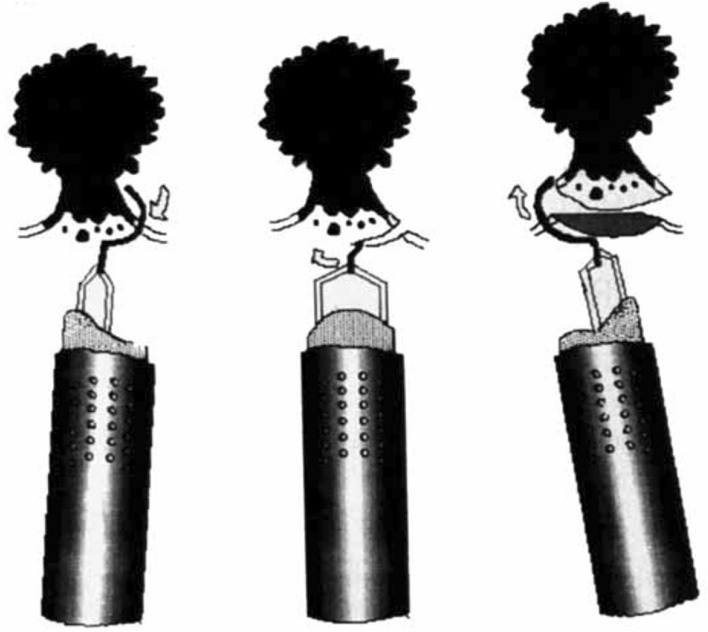
High-frequency wave resection was done by rotating electrode clockwise with handle while moving sheath to left side. Adapted from Kawada et al. J Urol 157 (6):2225–2226, with permission from the Journal of Urology

**Fig. 3:**
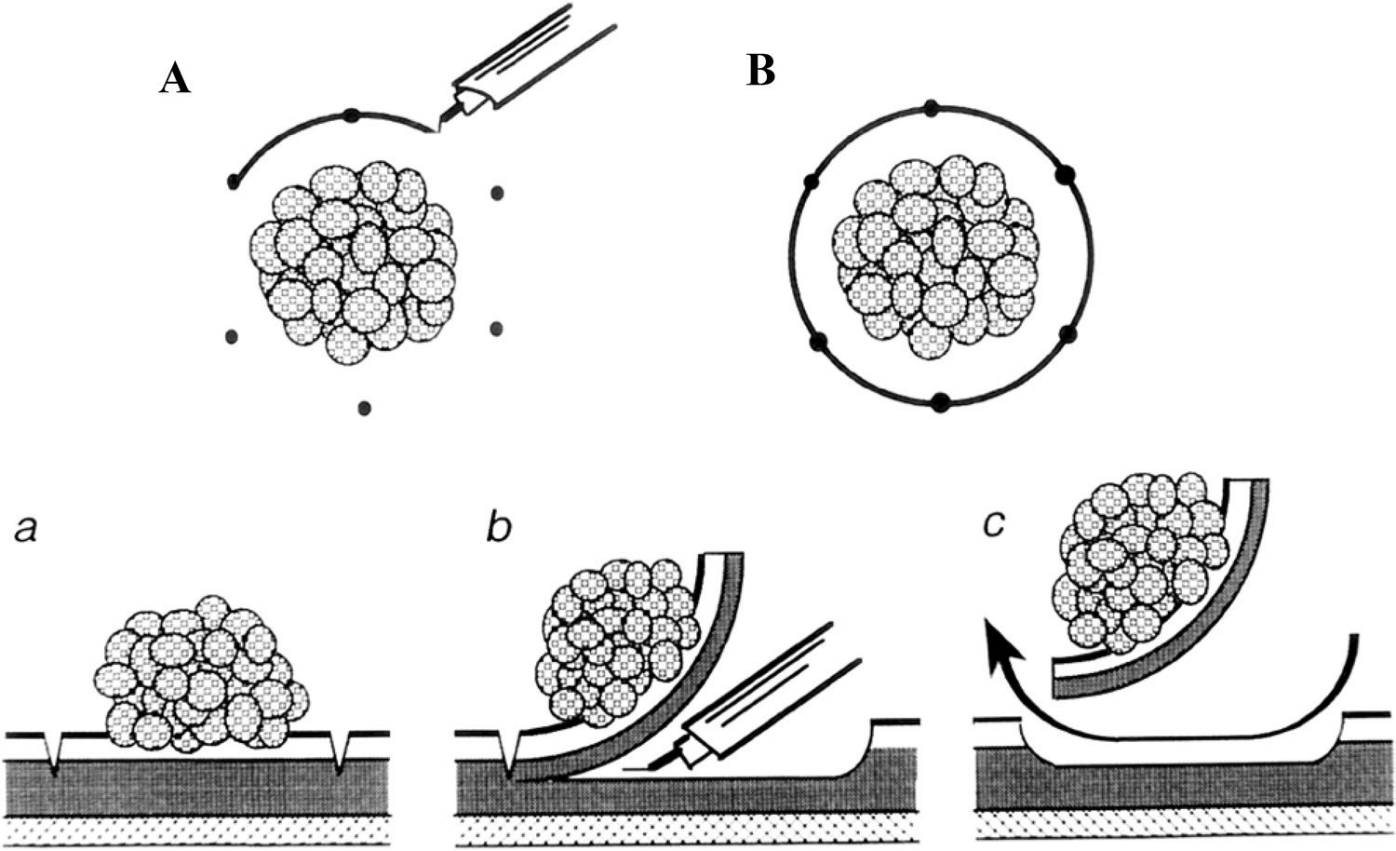
**A** and **B** View from above circular incision surrounding tumour. **a**, **b** and **c** Side view of serial level incisions through proper muscle. Adapted from Ukai et al. J Urol 163 (3):878–879, with permission from the Journal of Urology

The birth and early development of ERBT was largely pioneered by urologists from Japan. In 1980, Kitamura et al. published the first report on ERBT using a polypectomy snare through a transurethral resectoscope (Fig. 1) [8]. The polypectomy wire-loop snare was originally used to excise rectal polyps, but it was adapted for ERBT and bladder tumours ≤ 3 cm can be removed en bloc using this method [8]. In 1997, Kawada et al. reported the use of a tailor-made arched electrode for ERBT [9]. The bladder tumour was resected at the neck by swinging the sheath and rotating the arched resection electrode 180 degrees (Fig. 2) [9]. In 2000, Ukai et al. reported the basic steps of ERBT in a systematic manner, which forms the foundation of the ERBT procedure to this day [10]. Using a short curved needle electrode, a circular incision 5 mm from the tumour edge is first made, followed by incision underneath the bladder tumour at the detrusor muscle level; the bladder tumour specimen was then pinned on a foam board and sent for histological assessment (Fig. 3) [10]. In 2001, Saito reported the use of holmium laser for en bloc resection of bladder neck tumours and knife electrode for other bladder wall tumours, of which both modalities are commonly used nowadays [11]. This is also the first report providing T1 substaging in en bloc resected bladder tumour specimens [11]. Yanagisawa et al. later showed that ERBT could improve the diagnostic accuracy of T1 substage, and T1 substage was a significant predictive factor of disease progression [12, 13]. In 2005, thulium laser was introduced and was proposed to have a more precise incising and haemostatic effect than holmium laser [14]; its utility in performing ERBT was reported by Zhong et al. in 2010 [15]. Waterjet hydrodissection was first utilised in the gastroenterology field for endoscopic submucosal dissection [16]; such concept was adopted and applied in the urology field, and its safety and feasibility in performing ERBT was demonstrated by Nagele et al. in 2011 [17]. Subsequently, greenlight laser [18] has also been used in performing ERBT. In 2020, Hurle et al. reported the results of en bloc re-resection in patients with high-risk non-muscle-invasive bladder cancer (NMIBC); none of the patients experienced bladder perforation, and the recurrence rate at 3 months was only 3.85% [19].

### The biggest challenge in ERBT—tumour size

Removal of large bladder tumour has always been a challenge in ERBT. Teoh and Mostafid et al. published their experiences on routine implementation of ERBT in their clinical practices [20]. The overall technical success rate of ERBT regardless of tumour size was 73.3% [20]. When stratified according to tumour size, the technical success rates of ERBT were 84.3% and 29.6% for bladder tumour sizes of ≤ 3 cm and > 3 cm, respectively [20]. Several methods to facilitate extraction of large bladder tumours have been reported. In the study by Nagele et al. [17], a nylon retrieval bag was used to extract the tumour. In the paper by Naselli et al. [21], the authors suggested to make use of a morcellator telescope, and the 5 mm working channel is big enough to allow introduction of a 5 mm laparoscopic forceps to grasp and extract the tumour specimen. In 2018 [22, 23], Rapoport and Enikeev et al. proposed resecting and removing the exophytic part of the tumour by morcellation, followed by en bloc resection of the tumour base. Since the tumour base specimen remains intact, the histological assessment of the depth of tumour invasion and resection margins will not be affected. However, there may be an increased risk of tumour seeding upon morcellation and this should be carefully considered. Retrieval devices specifically designed for removal of large bladder tumours without morcellation are yet to be developed.

### The rebirth of en bloc resection through reminiscence

Since the introduction of ERBT back in 1980, there has been a gradual realisation of the critical steps of ERBT, and the energy modalities that can be used to perform a proper ERBT. Kramer and Herrmann et al. initiated the en bloc resection of urothelium carcinoma of the bladder (EBRUC) project, which is the first attempt ever to systematically research ERBT in a multi-centre setting [24]. In 2015, the group published a report comparing the safety and efficacy of laser (holmium and thulium) versus electrical (monopolar and bipolar) ERBT, and found that there was no statistically significant difference in complication rates, and recurrence rates at 3 months, 6 months and 12 months [24]. Although different energy modalities could have some differences in terms of their physical properties and technical details in execution, we must always bear in mind that for ERBT, surgical approach is primary, and energy modality is only secondary.

In 2020, Teoh et al. developed an international collaborative consensus statement of ERBT incorporating two systematic reviews, a two-round modified Delphi survey and a consensus meeting [25]. The “effectiveness” review evaluated the current evidence on ERBT compared to conventional TURBT, whereas the “uncertainties” review identified clinical and technical uncertainties of ERBT, which provided the basis for developing the statements for subsequent voting. A total of 103 statements were developed, and after the two-found Delphi survey and the consensus meeting, 99 of them (96%) reached consensus.

The key messages are as follows:

1. ERBT should always be considered for treating non–muscle-invasive bladder cancer (NMIBC).
2. ERBT should be considered feasible even for bladder tumours larger than 3 cm.
3. Number and location of bladder tumours are not major limitations in performing ERBT.
4. The planned circumferential margin should be at least 5 mm from any visible bladder tumour.
5. After ERBT, additional biopsy of the tumour edge or tumour base should not be performed routinely.
6. For the ERBT specimen, T1 substage, and circumferential and deep resection margins must be assessed.
7. It is safe to give a single dose of immediate intravesical chemotherapy, perform second-look transurethral resection, and give intravesical bacillus Calmette–Guérin (BCG) therapy after ERBT.
8. In studies of ERBT, both per-patient and -tumour analysis should be performed for different outcomes as appropriate.
9. Important outcomes for future ERBT studies were also identified. Specifically for ERBT, successful en bloc resection rate and resection margins should be reported. For the oncological outcomes, 3-month recurrence rate, 1-year recurrence and progression rates, and 5-year recurrence and progression rates are important outcomes to measure.

With a solid effort from global experts in ERBT, the consensus statement serves as a standard reference for urologists to practise ERBT and for researchers to conduct ERBT-related studies in the future.

### Increasing global interest and emerging evidence in ERBT

Over the past few decades, there has been an increasing interest in ERBT globally [26, 27]. In 2022, Yanagisawa et al. conducted a systematic review and meta-analysis including 13 randomised trials [28]. The authors found that ERBT was associated with a lower rate of bladder perforation (RR 0.13, 95% CI 0.05–0.34, *p* < 0.001) [28]. Detrusor muscle was also more likely to be present in the specimen following ERBT (RR 1.31, 95% CI 1.19–1.43,* p* < 0.001) [28]. However, 12-month (RR 0.98, 95% CI 0.76–1.26, *p* = 0.86) and 24-month recurrence (RR 0.83, 95% CI 0.55–1.22, *p* = 0.35) were similar between ERBT and TURBT [28].

Subsequently, results from three other randomised trials comparing ERBT with TURBT have been reported. In the single-centre randomised trial by Gallioli and Breda et al. [29], a total of 300 patients were randomised to receive either ERBT (monopolar, bipolar or thulium laser) or TURBT (monopolar or bipolar). The rate of detrusor muscle presence for ERBT was found to be non-inferior to TURBT (94% vs 95%, *p* = 0.8). T1 substaging was more feasible in the ERBT group (100% vs 80%, *p* = 0.02). Peri-operative outcomes including complications rates, catheterisation time and hospital stay were similar between the two groups, and the recurrence rate at median follow-up of 15 months was 18% for TURBT and 13% for ERBT (*p* = 0.16). In the multi-centre randomised trial by D’Andrea and Shariat et al. [30], a total of 399 patients were randomised to receive either ERBT or TURBT. ERBT resulted in a higher rate of detrusor muscle presence when compared to TURBT (80.7% vs 71.1%, *p* = 0.01). Operative time was similar between the two groups, but the ERBT group had a lower rate of bladder perforation (5.6% vs 12%) than the TURBT group. With a median follow-up of 13 months, recurrence rates were 18.4% in the ERBT group and 16.7% in the TURBT group (*p* = 0.6). In the multi-centre randomised trial by Teoh et al. [31], a total of 350 patients were randomised to receive either bipolar ERBT or bipolar TURBT. Regarding the primary outcome, the 1-year recurrence rates were 28.5% (95% CI 18.4–37.4%) in the ERBT group, and 38.1% (95% 28.4–46.5%) in the TURBT group (*p* = 0.007) [31]. The 1-year progression rates were 0% in the ERBT group, and 2.6% (95% CI 0–5.5) (*p* = 0.065) in the TURBT group [31]. Operative time was longer in the ERBT group (median 28 vs 22 min, *p* < 0.001), but detrusor muscle sampling rates, hospital stay and 30-day complications were similar between the two groups [31]. To date, the randomised study by Teoh et al. is the only clinical trial that demonstrated a significant benefit in recurrence rate [31], and the overall evidence regarding its potential superiority is still controversial. An individual patient data meta-analysis is currently underway, and hopefully, it can provide more insights regarding the true value of ERBT.

### Evolving concepts and future directions of ERBT

Most urologists would agree that ERBT has two main goals and potential benefits, (1) to ensure complete resection of bladder tumour, and (2) to minimise the risk of tumour seeding by extracting the bladder tumour in one piece. While the term ‘en bloc’ sounds appealing, its literal definition of ‘removal in one piece’ does not reflect the potential benefits of the procedure completely. For large NMIBC, there is a higher chance of residual disease following TURBT, and being able to ensure complete resection of bladder tumour is probably more important than a mere removal of bladder tumour in one piece. In case of muscle-invasive bladder cancer (MIBC), ensuring proper local staging of the disease is most important, and whether you remove the bladder tumour in one piece or multiple pieces has minimal implications in the subsequent management of the disease. In addition, ERBT can possibly achieve maximal transurethral resection, which may be helpful in optimising subsequent treatment such as radical cystectomy and trimodality therapy [32–34]. Therefore, for large bladder tumours, even if we cannot extract the tumour in one piece, it might still be beneficial to optimise the resection quality by resecting the bladder tumour with the usual en bloc resection principles. This forms the basis for the evolving concept of modified ERBT for large bladder tumours.

**Fig. 4:**
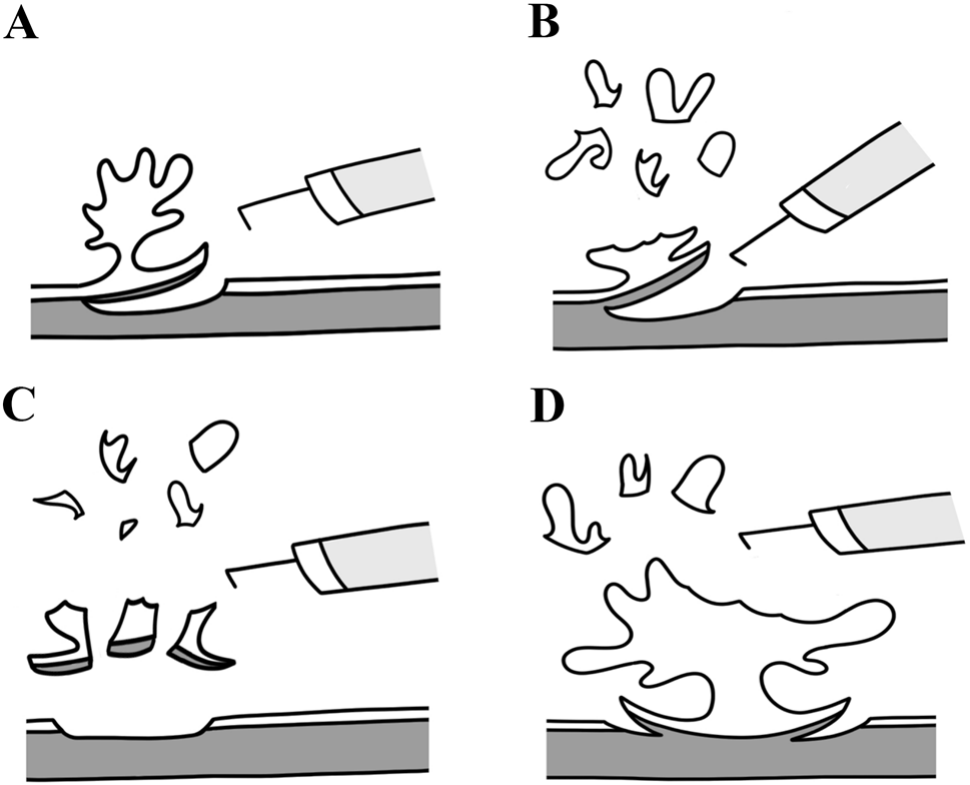
**A** Classical en bloc resection of bladder tumour (ERBT). **B** Modified ERBT by resecting the exophytic part of bladder tumour in a piecemeal manner, followed by en bloc resection of the tumour base. **C** Modified ERBT by resecting the exophytic part of bladder tumour in a piecemeal manner, followed by en bloc resection of the tumour base, and removal of tumour base specimen in multiple pieces. **D** Transurethral resection of bladder tumour by following the ERBT principles, i.e. define the resection margins and incise down to the normal detrusor muscle layer circumferentially, work towards the central part of the tumour base from lateral to medial and from normal to abnormal, and finally resect the bladder tumour in a piecemeal manner by constantly taking reference from the pre-defined normal detrusor muscle layer circumferentially

The general principle of modified ERBT is to resect large bladder tumours as en bloc as reasonably achievable. In the most ideal case, the whole bladder tumour can be resected and removed in one piece (Fig. 4A). However, in situations when a true ERBT cannot be achieved, modified ERBT can be considered and there are several ways to achieve this (Fig. 4B–D). First, we can resect the exophytic part of the bladder tumour in piecemeal manner, followed by en bloc resection of the tumour base (Fig. 4B). Likewise, we can excise the main bulk of the exophytic part of the bladder tumour and remove it by morcellation, followed by en bloc resection of the tumour base. For even larger tumours where en bloc removal of the tumour base is not feasible, one can consider removing the tumour base in multiple pieces (Fig. 4C). In the most technically challenging situations where conventional TURBT is the only option, one should still try to follow the en bloc resection principles (Fig. 4D). Define the resection margins and incise down to the normal detrusor muscle layer circumferentially, work towards the central part of the tumour base from lateral to medial and from normal to abnormal, and finally resect the bladder tumour in a piecemeal manner by constantly taking reference from the pre-defined normal detrusor muscle layer circumferentially (Fig. 4D). A phase 2 trial on modified ERBT focusing on patients with bladder tumours > 3 cm is currently under way [35]. The study aims to recruit 30 patients, and it has a composite primary outcome of (1) Complete resection for NMIBC (defined as absence of malignancy upon second-look TURBT), and (2) Proper staging for MIBC (defined as the detection of MIBC upon the first modified ERBT). Hopefully, the study will be able to provide valuable information on whether such modified approach has any potential benefits in treating large bladder tumours.

As high-quality evidence on ERBT is emerging, it is also important to assess its feasibility and generalizability in everyday clinical practice from a global perspective. The global ERBT registry has been launched and it aims to recruit 2000 patients in total [36]. This registry aims to collect real-world data on ERBT, and to provide insights on important questions that are difficult to answer with randomised trials. For example, in case of clear resection margins but absence of detrusor muscle in the ERBT specimen, is second-look TURBT still needed? Should second-look TURBT be offered in case of positive resection margins in the ERBT specimens? Can clear resection margins be achieved at all in case of early muscle-invasive bladder cancer, and if so, is radical treatment still needed? With a global collaborative effort, this registry will allow us to have a much better understanding about the role of ERBT in bladder cancer and how to optimise it further.

Surgical training is paramount to a proper and successful dissemination of the ERBT technique. A porcine bladder TURBT model has been developed and validated, and it is useful for ERBT training [37]. Currently, there is no data regarding the learning curve of ERBT, but the authors believe that a minimum of 20 cases is needed for successful adoption of the technique.

## Conclusions

**Fig. 5:**
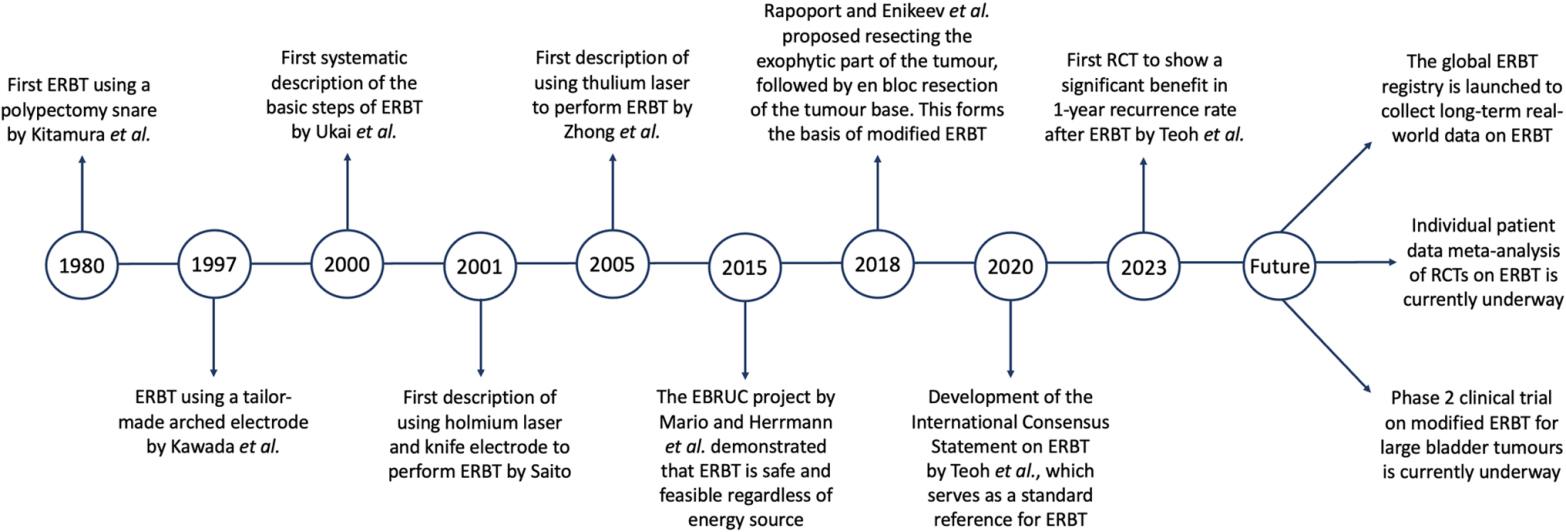
Timeline on the history, development and future directions of ERBT. *ERBT* en bloc resection of bladder tumour, *EBRUC* en bloc resection of urothelium carcinoma, *RCT* randomised controlled trial

This review paper summarises the history, development and future directions of ERBT, which represents the hard work of several general generations of urologists from different parts of the world (Fig. 5). Throughout the past few decades, we have come to understand a lot more about the ERBT procedure, and it enhanced the spectrum of endoscopic approaches in treating NMIBC. Recently, high-quality evidence has been emerging rapidly, and at this juncture, an individual patient data meta-analysis would be instrumental in defining the true value of ERBT in treating NMIBC. A modified ERBT approach might be the way forward to treat large bladder tumours but its efficacy remains to be defined. While the evidence on ERBT is promising, there are still many important clinical questions to be answered, and the global ERBT registry will be able to provide valuable insights on this.
